# Effect of Gellan Gum and Xanthan Gum Synergistic Interactions and Plasticizers on Physical Properties of Plant-Based Enteric Polymer Films

**DOI:** 10.3390/polym12010121

**Published:** 2020-01-05

**Authors:** Na Zhang, Xiaohui Li, Jing Ye, Yucheng Yang, Yayan Huang, Xueqin Zhang, Meitian Xiao

**Affiliations:** 1 College of Chemical Engineering, Huaqiao University, Jimei Road, Jimei District, Xiamen 361021, Fujian, China; xiaohuili0715@163.com (X.L.); yejenny@hqu.edu.cn (J.Y.); yangyc@hqu.edu.cn (Y.Y.); yyhuang@hqu.edu.cn (Y.H.); xqzhang2009@hqu.edu.cn (X.Z.); 2 Xiamen Engineering and Technological Research Center for Comprehensive Utilization of Marine Biological Resources, Jimei Road, Jimei District, Xiamen 361021, Fujian, China

**Keywords:** plant-based enteric polymer films, hard capsules, synergistic interactions, gelling agent, plasticizer, mechanical property, barrier property

## Abstract

The mechanical and barrier properties of plant-based enteric polymer films were enhanced by synergistic interactions between binary gum mixtures and adding plasticizers. The results indicated that the best ratio of gellan gum (GG) and xanthan gum (XG) was 7:3 by comparing tensile strength, tensile elongation, transmittance, and water vapor permeability of plant-based enteric polymer films and rheological properties of solutions. Polyethylene glycol 400 (PEG-400) was an effective plasticizer in improving plasticity and water vapor barrier property of the plant-based enteric polymer film. Rheology measurement and different characterization methods, including Fourier transform infrared spectroscopy, thermogravimetric analysis, differential scanning calorimetry, X-ray diffraction, and scanning electron microscopy, were used to explain interactions between GG and XG as well as PEG-400 and components of the film. The new mixed system, composed of GG/XG mixture with ratio of 7:3 as a novel gelling agent and PEG-400 as a plasticizer, was applied to prepare plant-based enteric hard capsules, which have potential applications in medicines and functional food preparations.

## 1. Introduction

Generally, plant-based enteric polymer films are designed to be insoluble in gastric juice and soluble in intestinal juice and used to produce plant-based enteric hard capsules [1]. At present, the method of preparing plant-based enteric hard capsules is to coat the surfaces of capsules with enteric-based materials [2]; however, this method requires an additional coating process, and thus raises production costs [3]. Recently, plant-based enteric hard capsules without the requirement of a coating process have been reported. However, these studies mainly focused on the formulation and technological conditions of preparing enteric hard capsules without examining mechanical and barrier properties of the plant-based enteric polymer films [4,5,6,7,8]. Plant-based enteric hard capsules can passage through the stomach and dissolve in the intestine, and kept intact in the stomach. Therefore, mechanical and barrier properties of the plant-based enteric polymer films are very important, which directly affect the disintegration properties and storage stability of capsules [9]. However, to the best of our knowledge, information on mechanical and barrier properties of plant-based enteric polymer films is hitherto rare.

Currently, the study of mechanical and barrier properties on edible films has been extensively reported, which can provide a valuable reference for the study of plant-based enteric polymer films, as plant-based enteric polymer films using plant gum as raw material are similar to edible films. Synergistic interactions between gum mixtures have attracted wide attention in food industry because they can improve rheological, mechanical, and barrier properties of products [10]. Frequently, xanthan gum (XG) was mixed with other biopolymers to change the viscosity of biopolymers, due to the synergistic interaction between them [10,11,12,13]. Furthermore, XG can improve mechanical properties and water vapor permeability of protein edible films [14]. Kurt found that a biodegradable edible film could be prepared by synergistic interactions between XG and locust bean gum, and the result showed mechanical and barrier properties of the films were changed when different mixing ratios of XG and locust bean gum and glycerol as a plasticizer were added [15].

Generally, plasticizers can improve flexibility, reduce brittleness and strongly affect physicochemical properties of the film [16]. For most of the carbohydrates, water is the “natural” plasticizer but volatile [17]. Moreover, a variety of non-volatile plasticizers were used in edible films in order to improve physicochemical properties of the films such as glycerol [9,18,19], sorbitol [20,21], polyethylene glycol (PEG) [22], xylitol [23], mannitol [23], or low-molecular-weight sugar [24]. Particularly, plasticizers with diverse hydrophilic hydroxyl groups are compatible with the polysaccharides constituted the film network, thus improve mechanical and barrier properties of the films [25].

Therefore, the aims of this work were to develop a novel gelling agent composed of gellan gum (GG) and XG for preparation of plant-based enteric polymer films and to evaluate the effect of different ratios of GG and XG and types and molecular weights of plasticizers on plant-based enteric polymer films’ properties. The mechanical, optical, and barrier properties of plant-based enteric polymer films were evaluated by analyzing the films’ tensile strength (TS), tensile elongation (TE), transmittance, and water vapor permeability (WVP). In addition, the interactions between GG and XG as well as plasticizer and components of the film were assessed by rheology measurement, Fourier transform infrared spectroscopy (FTIR), thermogravimetric analysis (TGA), differential scanning calorimetry (DSC), X-ray diffraction (XRD), and scanning electron microscopy (SEM). By this work, plant-based enteric hard capsules can be prepared by a novel gelling agent, comprising GG and XG, and have potential applications in medicines and functional food preparations

## 2. Materials and Methods

### 2.1. Materials

Commercially available food-grade hydroxypropyl methylcellulose (HPMC) (HT-E15), XG (*M*_w_ = 500,000–600,000), GG (*M*_w_ = 400,000–600,000), potassium citrate, and calcium chloride were purchased from Shandong Rutocel Co., Ltd. (Taian, China). Sodium alginate (*M*_w_ = 98,000; G/M = 1), fructose (*M*_w_ = 180.15) and sorbitol were provided by Shanghai Macklin Biochemical Technology Co., Ltd. (Shanghai, China). Glycerol, beeswax, and PEG were supplied by Sinopharm Chemical Reagent Co., Ltd. (Shanghai, China).

### 2.2. Methods

#### 2.2.1. Solution Preparation

A solution was prepared by dissolving 1.0% (*w/w*) GG and XG mixture of a ratio 7:3 in distilled water containing 0.3% (*w/w*) potassium citrate and 1.0% (*w/w*) PEG-400 at 60 °C. After the solution was heated to 80 °C, 9% (*w/w*) HPMC and 0.7% (*w/w*) sodium alginate were added to it with stirring; then, the solution was kept at 80 °C for 1 h with moderate agitation. After all the chemicals were dissolved, the solution was cooled to 50 °C and degassed using a vacuum pump at −0.04 MPa for 2 h. Then, the solution was stored in a water bath at 50 °C for 12 h.

#### 2.2.2. Film and Hard Capsule Preparation

The solution (10.0 g) was poured and gelled on a flat glass (10 cm × 10 cm), and then dried in a constant temperature and humidity oven at 28 °C with RH of 60% for 3 h. The thicknesses of all films were measured to be 0.10 mm with about 5% standard deviation by a micrometer after drying.

Plant-based enteric hard capsules were prepared using the well-established method of dipping stainless steel mold pins (cylindrical: cap, 7.5 ± 0.02 mm; body,7.1 ± 0.02 mm) into the solution followed by dipping into calcium chloride solution (5% (*w/w*)) for 30s and dried for 3 h [26].

#### 2.2.3. Sample Preparation

The blank film was made of 9% (*w/w*) HPMC, 0.7% (*w/w*) sodium alginate, 0.4% (*w/w*) GG, and 0.3% (*w/w*) potassium citrate. Plasticized films were prepared by adding 1.0% (*w/w*) different plasticizers including glycerol, beeswax, fructose, sorbitol, and PEG to the blank films.

The powder mixture was prepared by mixing 9% (*w/w*) HPMC, 0.7% (*w/w*) sodium alginate, 0.4% (*w/w*) GG, and 0.3% (*w/w*) potassium citrate together. The GG films were prepared by 9% (*w/w*) HPMC, 0.7% (*w/w*) sodium alginate, 0.4% (*w/w*) GG, and 0.3% (*w/w*) potassium citrate. The GG films blended XG was named as GG/XG films. And the GG/XG films added plasticizer PEG-400 was named as PEG films.

### 2.3. Characterization

#### 2.3.1. Mechanical Properties

The TS and TE analysis of film were performed by using a 104B computer controlled electromechanical universal testing machine (Shenzhen Wance testing machine Co., Ltd., Shenzhen, China). Film specimens were measured according to ASTM standard method D882 with some modifications [27]. Films were cut into dumbbell strips (width: 10 mm; gauge length: 50 mm), which were placed into the testing system that was set at a grip distance of 80 mm and a grip speed of 2 mm/min.

#### 2.3.2. WVP

The WVP of films was measured according to the ASTM standard method E96-95 [28], and the values of WVP were calculated by Equation (1) [29]:

(1) $$\mathrm{WVP}=\frac{\Delta m}{\Delta t\cdot A\;}\times \frac{X}{\Delta p}$$

where Δ*m*/Δ*t* is the weight of moisture gain per unit time (g/s), *A* is the area of the exposed film surface (m^2^), *X* is the average thickness of the film (mm), and Δ*p* is the water vapor pressure difference between the two sides of the film (Pa).

#### 2.3.3. Optical Properties

Transmittance of films was determined according to the method described by Gontard [30] using an UV-1800 ultraviolet–visible spectrophotometer (Shanghai Mapada instrument Co., Ltd., Shanghai, China).

#### 2.3.4. Rheological Measurements

The rheological measurements were performed with a DHR-2 rheometer (TA Instruments, New Castle, DE, USA). The GG/XG mixed solutions were analyzed by using a cone-and-plate geometry (diameter: 40 mm, cone angle: 1°) at a gap distance of 1.0 mm. The measurements were carried out by shear rate from 0.1 to 600 s^−1^ at 50 °C. The samples were measured with a range of temperature sweep from 30 °C to 90 °C at the rate of 0.5 °C/min. The frequency was fixed at 2.0 Hz and the strain amplitude was 2%. The storage modulus (G’) and loss modulus (G”) were obtained by the TRIOS software (v4.3.1, TA Instruments, New Castle, DE, USA) of TA Instruments. The gelling temperature and melting temperature values of gel could be confirmed by the crossover point of G’ and G’’ curve during the heating and cooling processes [31]. The loss tangent was the ratio of G’’ to G’, initial loss tangent (tanδ_0_) was the loss tangent at the lowest temperature, and gelation rate was the change rate of G’ with time [32,33].

#### 2.3.5. FTIR

FITR spectra of power mixture, GG films, GG/XG films, and PEG films were recorded on a FTIR-84 spectrometer (Shimadzu, Kyoto, Japan) from 400 to 4000 cm^−1^ with 32 scans.

#### 2.3.6. TGA

The thermal stability of samples was evaluated by a DTG-60H thermogravimetric analyzer (Shimadzu, Kyoto, Japan). The sample (3–10 mg) was heated from 20 to 600 °C at a heating rate of 10 °C/min under argon atmosphere with a flow rate of 50 mL/min.

#### 2.3.7. DSC

DSC experiments of samples were performed on a DSC 200 differential scanning calorimetry instrument (NETZSCH-Gerätebau GmbH, Selb, Germany). The samples (3–5 mg) were heated from −20 to 300 °C at 10 °C/min under argon atmosphere with a flow rate of 50 mL /min.

#### 2.3.8. XRD

XRD patterns were obtained by a MiniFlex600 X-ray diffractometer (Rigaku, Tokyo, Japan). Cu Ka (1.54056 Å) was selected as the monochromic energy radiation. The diffraction angle (2θ) was scanned from 10° to 70° at a rate of 10°/min at 25 °C.

#### 2.3.9. SEM

The cross-section morphologies of the film samples were obtained using a SU8000 scanning electron microscopy (Hitachi, Tokyo, Japan). Samples were fastened to a sample holder and sputtered with a gold-palladium layer using a magnetron ion sputter device (MSP-2S) and then observed by SEM at a low accelerating voltage (3 kV).

## 3. Results and Discussion

### 3.1. Properties of Films with Different GG/XG Ratios

**Figure 1:**
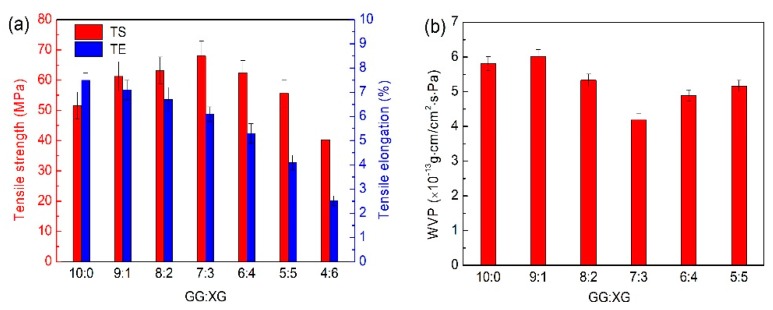
Effects of GG/XG mixing ratio on (**a**) mechanical properties and (**b**) barrier properties of films.

To modify the mechanical properties and WVP of the plant-based enteric polymer films, XG was added to the films and the mixture of GG and XG was used as a novel gelling agent. The increase of XG led to the gradual decrease of TE, and although the TS of films increased from 51.6 MPa to the maximum of 68.0 MPa at a GG/XG mixing ratio of 7:3, they gradually decreased to 40.2MPa (Figure 1a). At the same time, WVP of the film at a GG/XG mixing ratio of 7:3 (4.18 × 10^−13^ g·cm/cm^2^·s·Pa) in Figure 1b was the lowest, suggesting the water vapor barrier property of the film was the best. Our study indicated that GG and XG might have a synergistic interaction and the optimum ratio of GG/XG was 7:3.

### 3.2. Rheological Analysis of Solution

**Figure 2:**
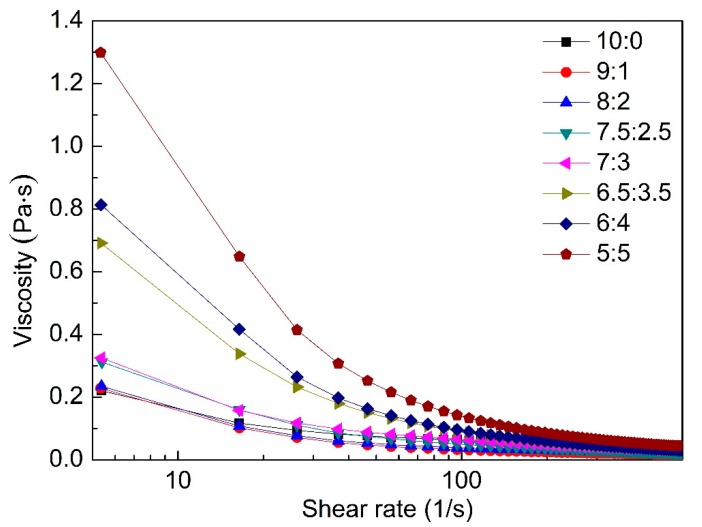
Shear viscosity of solutions at different GG/XG mixing ratios.

To study the effect of XG on the rheological properties of GG/XG mixture solutions and evaluate the synergistic interaction between GG and XG, the solutions of different GG/XG ratios (10:0, 9:1, 8:2, 7.5:2.5, 7:3, 6.5:3.5, 6:4, 5:5) were prepared. The plots of viscosity versus shear rate of the solutions at different GG/XG mixing ratios were showed in Figure 2. The viscosity of all the solutions decreased with the increase of shear rate, and then the viscosity kept almost constant, indicating that the GG/XG mixed solutions were non-Newtonian fluids. Addition of XG increased the shear-thinning behavior of the GG/XG mixed solutions [12]. The viscosities of GG/XG mixed solutions increased from 0.22 to 1.3 Pa·S with addition of XG from 0% to 50% at shear rate of approximately 5.4 s^−1^. When GG/XG mixing ratio was higher than 7:3, the viscosity of the mixtures significantly increased at low shear rate. The result showed a synergistic interaction formed between GG and XG [34,35].

The effect of XG on rheological properties was further studied by dynamic rheology; the results are shown in Table 1. Gelling temperature and melting temperature of the solutions changed depending on the ratio of XG. When the GG/XG mixing ratio was 7:3, gelling temperature and melting temperature of the solution reached the maximum values of 82 °C and 72 °C, respectively, indicating that GG and XG had the synergistic interaction. At the point of GG/XG mixing ratio 5:5, gelling temperature and melting temperature of the solution were undetectable. The reason might be attributed to the excessive content of XG, which had the property of weak gelling interfering with the formation of network structure of gel [11]. The gelation rate of all the solutions obviously increased with addition of XG, and then it slowly increased when the GG/XG ratio reached 7:3. The change of gelation rate indicated that there was synergistic interaction between GG and XG, resulting in the change of their structure [33]. The tanδ_0_ of the solution with the GG/XG mixing ratio of 7:3 reached the minimum value 0.2114, and exhibited the best elasticity in all solutions [36].

### 3.3. Type of Plasticizer

**Figure 3:**
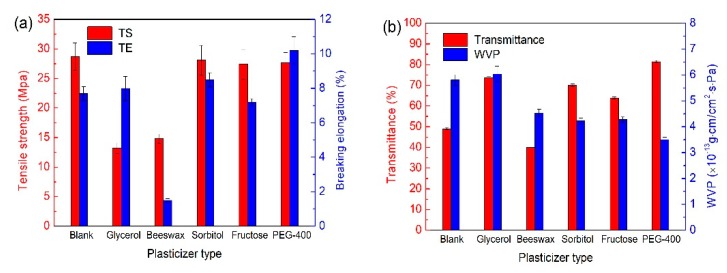
Effects of plasticizer types on (**a**) mechanical properties and (**b**) barrier properties of films.

The mechanical properties of the blank film and five plasticized films were shown in Figure 3a. As can be seen, the glycerol and beeswax plasticized films had obviously lower TS than the other four films. However, no obvious differences were discovered in TS of these four films. In addition, the glycerol, sorbitol, and PEG-400 plasticized films had higher TE than the blank; among them, the PEG-400 plasticized film had the highest TE. The results indicated that PEG-400 is an effective plasticizer in improving mechanical properties of films. However, Yang found that the glycerol plasticized gellan films were more stretchable than the PEG plasticized gellan films [16]. The difference might be caused by the different components of gellan films [37].

The transmittances of different plasticized films were shown in Figure 3b. Glycerol, fructose, sorbitol, and PEG-400 plasticized films had high transmittance of 73.6%, 63.9%, 70%, and 81.4%, respectively, much higher than that of the blank (<50%). The reason might be attributed to the structural homogeneity of plasticizer and polysaccharides constituted the film network [38]. Figure 3b showed that the WVPs of beeswax, sorbitol, fructose, and PEG-400 plasticized films were much lower than that of the blank, suggesting that hydrophilic hydroxyl groups in beeswax, sorbitol, fructose, and PEG-400 were inclined to develop hydrogen interactions with polysaccharides composed of the film replacing interactions between polysaccharides [39,40]. Therefore, PEG-400 plasticized films (3.50 × 10^−13^ g·cm/cm^2^·s·Pa) had the lowest WVP and the best barrier properties.

### 3.4. Molecular Weight of PEG

**Figure 4:**
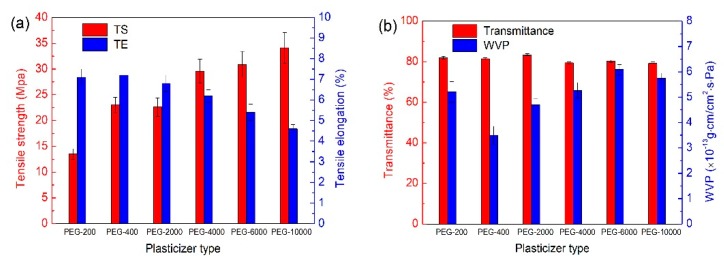
Effects of molecular weight of plasticizer PEG on (**a**) mechanical properties and (**b**) barrier properties of films.

The effect of molecular weight of PEG on mechanical properties and barrier property was discussed in Figure 4. The TS of films were increased while TE decreased with the increase of molecular weight of PEG. PEG-200 and PEG-400 endowed plasticized films more stretchable than others in Figure 4a. The reason might be that low molecular weight of PEG could be more easily inserted between polysaccharide chains constituted the film network, producing the cross-linking between polysaccharide and PEG, which would reduce the free volume and the mobility of polysaccharide segments, decreasing the mechanical strength of the film, enhancing its extensibility [9]. Figure 4b shows that the molecular weight of PEGs had no influence on the transmittance of films. However, the WVP was affected by the molecular weight of PEG. With the increase of molecular weight of PEG from approximately 200 to 10,000, WVP of the films remarkably decreased from 5.21 × 10^−13^ g·cm/cm^2^·s·Pa to 3.50 × 10^−13^ g·cm/cm^2^·s·Pa and then gradually increased to 6.09 × 10^−13^ g·cm/cm^2^·s·Pa. Among them, WVP of PEG-400 plasticized film was the lowest, which indicated that the PEG-400 had the greater hydrogen interactions with polysaccharides composed of the film than other plasticizers [40].

### 3.5. FTIR Analysis of Films

**Figure 5:**
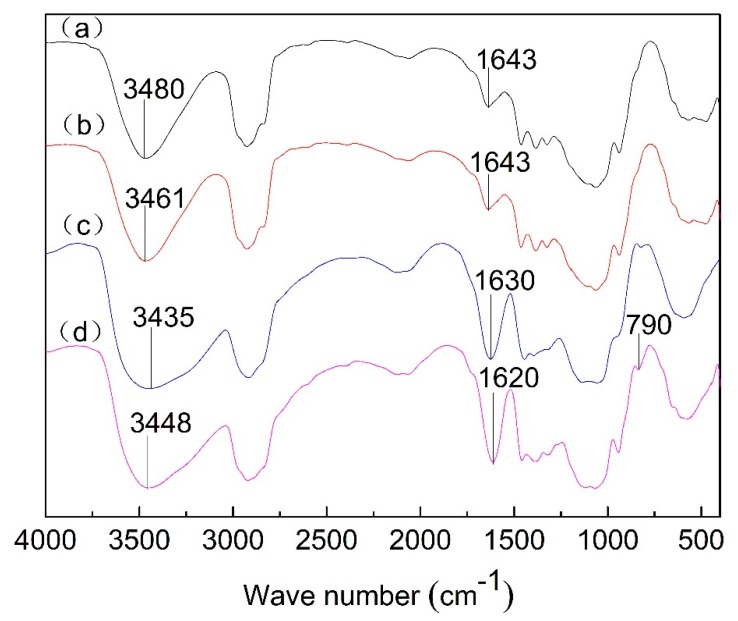
FTIR spectra of (**a**) power mixture, (**b**) GG film, (**c**) GG/XG film, and (**d**) PEG film.

The interaction between polysaccharides composed of the film can be investigated by the shift of the peak position of their main groups in FTIR spectra [41], and the results were shown in Figure 5. The strong absorption bands centered at 3700–3200 cm^−1^ attributed to the stretching of intermolecular hydrogen bonds showed significant changed. Compared to power mixture, the absorption band at 3480 cm^−1^ was broader and shifted to lower wave numbers for GG film, GG/XG film and PEG film. The shift was caused by hydrogen bonding as a result of the additional OH groups provide by GG, XG, and PEG. This band of 3461 cm^−1^ in the GG film shifted to 3435 cm^−1^ in the GG/XG film, suggesting an increase in intermolecular hydrogen bonding between GG and XG, which indicated synergistic interaction between GG and XG [42]. The band of 1643 cm^−1^ was assigned to carbonyl stretching vibrations and the shift of carbonyl band implied that hydrogen bonding occurred between GG and XG molecules in films [43]. This band of 1630 cm^−1^ in the GG/XG film shifted to 1620 cm^−1^ in the PEG film, indicating an increase in intermolecular hydrogen bonding between plasticizer and polysaccharides composed of the film. In addition, a signal that appeared at 790 cm^−1^ in PEG film confirmed the existence of long carbon chain belonging to plasticizer PEG-400. Compared to power mixture, new absorption bands had not been found except band of 790 cm^−1^ belonged to PEG-400. Therefore, the absence of new absorption bands indicated that the synergistic interaction between GG and XG and the intermolecular interaction between plasticizer and the polysaccharides composed of the film were physical cross-linking.

### 3.6. Thermal Analysis of Films

**Figure 6:**
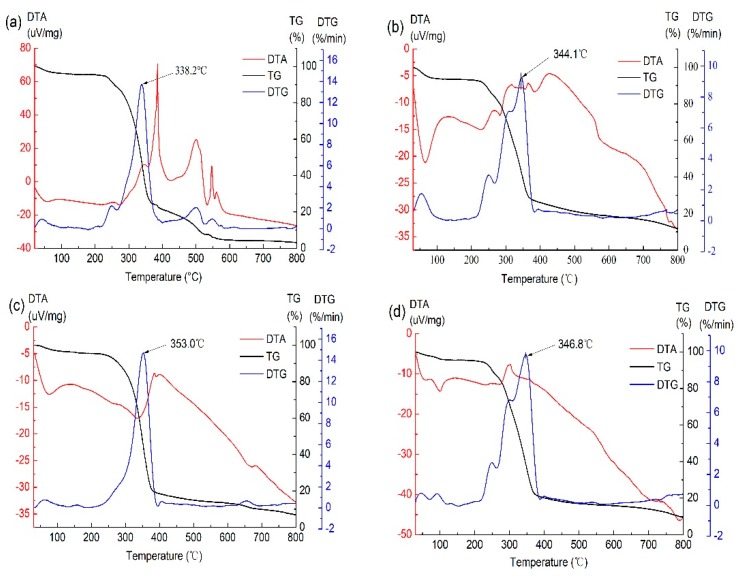
TG, DTG, and DTA curves of (**a**) powder mixture, (**b**) GG film, (**c**) GG/XG film, and (**d**) PEG film.

Thermal analysis can provide information about stability, compatibility, degradation and glass transition occurring in biomaterials during thermal change [44], and the results were shown in Figure 6. The degradation temperature of powder mixture, GG film, GG/XG film and PEG film was 338.2, 344.1, 353.0, and 346.8 °C, respectively. Addition of plasticizer in film, characterized by a lower degradation temperature, reduced the thermal stability of film which was due to the intermolecular interaction between plasticizer and components of the film. The increased thermal stability of GG/XG film was attributed to the synergistic interaction between GG and XG. The differential thermal analysis (DTA) curve of the powder mixture showed five obvious peaks, indicating the five components in the powder mixture were dispersed and no interactions between those components. The existence of interactions in GG/XG film and PEG film was confirmed by a unique broad peak in the DTA curve for multiple compounds [45].

The activation energy (E) values of the degradation processes were showed in Table 2. The E value increased with the addition of XG to the GG film, which decreased when plasticizer PEG-400 was added to the GG/XG film. This result was consistent with those of the differential thermogravimetry (DTG). Glass transition temperature (*T*_g_) values of different films were significantly affected by XG and plasticizer PEG-400 according to DSC measurements reported in Table 2. The *T*_g_ of GG/XG film with XG (213.0 °C) was much higher than that of GG film (150.1 °C), displaying the synergistic interaction between GG and XG. The *T*_g_ of PEG film (159.5 °C) was lower than that of GG/XG film. The result was consistent with those obtained by the DTA analysis, suggesting that the thermal stability of the films was reduced by adding plasticizer in the film formulation.

### 3.7. XRD of Films

**Figure 7:**
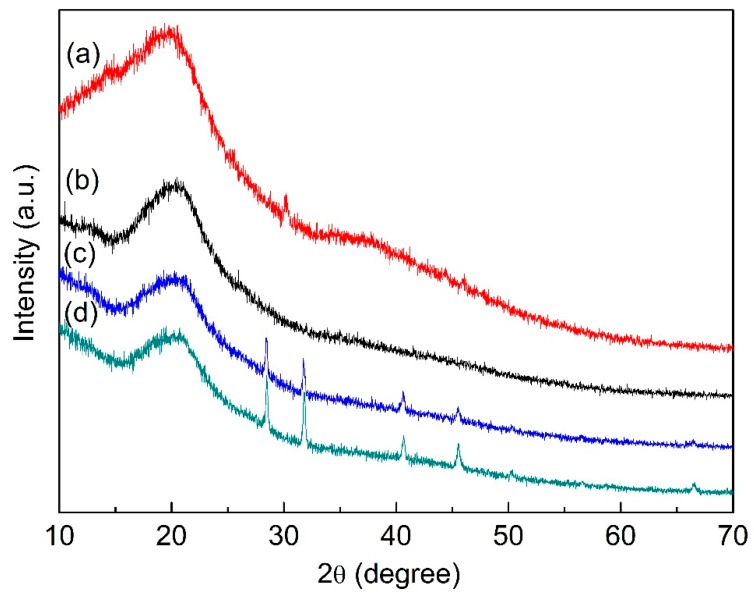
XRD patterns of (**a**) XG powder, (**b**) GG film, (**c**) GG/XG film, and (**d**) PEG film.

All films showed a broad peak at approximately 2θ = 20°, indicating a typical noncrystalline structure in Figure 7. The disappearance of the broad peak at approximately 2θ = 38° (Figure 7a) in the GG/XG film confirmed the synergistic interaction between GG and XG (Figure 7c).

The presence of four sharp peaks at 2θ = 28.4°,31.74°, 40.66°, and 45.53° was probably due to the presence of inorganic salt–potassium citrate in GG/XG film and PEG film (Figure 7c,d) [10,46]. However, the four sharp peaks were not observed in the GG film containing inorganic salt, which indicated that inorganic salt played an important role in the gelation process of GG (Figure 7b). The inorganic ions had specific site binding to GG, which had an effect on the aggregation and gel formation of GG molecules [16]. The same four sharp peaks were observed when XG was added to the GG film (Figure 7c). However, it is evident that the interaction between GG and XG by hydrogen bonds was stronger than that of GG and inorganic salt. For PEG film (Figure 7d), the intensity of the four sharp peaks increased, which indicated that hydroxyl groups in plasticizer PEG-400 easily developed hydrogen bonds between PEG and XG replacing the intermolecular interactions between XG chains by adding inorganic salt [47].

### 3.8. SEM of Films

**Figure 8:**
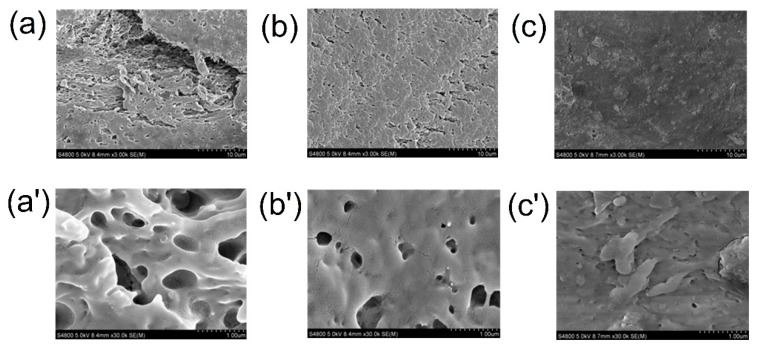
SEM micrographs of the cross-section of (**a**,**a’**) GG film, (**b**,**b’**) GG/XG film and (**c**,**c’**) PEG film. Cross section (**a–c**) viewed at a magnification of 3000× and cross section (**a’–c’**) viewed at a magnification of 30,000×, respectively.

To better understand the influence of XG and plasticizer on the structural characteristic of films, SEM was performed to observe the cross-sections of all prepared films. Figure 8a,a’ show the cross-section micrographs of the GG film viewed at a magnification of 3000× and 30,000×, respectively, in which many pores and cracks were observed. Obviously, the mechanical properties and WVP of the GG film needed to be improved. By comparison, a more regular, compact and less pored structure was observed of the GG/XG film, suggesting the synergistic interaction existed between GG and XG in Figure 8b,b’. Furthermore, a compact structure without any pores was found in the PEG film (Figure 8c,c’). This result illustrated that plasticizer PEG-400 could easily insert between polysaccharide chains constituted the film network, produce cross-linking and then fill in pores of the films [9]. Therefore, the PEG film had better mechanical properties and lower water vapor permeability. The results were also in good agreement with those of FTIR spectra and XRD patterns.

### 3.9. Photograph of Plant-Based Enteric Hard Capsules

**Figure 9:**
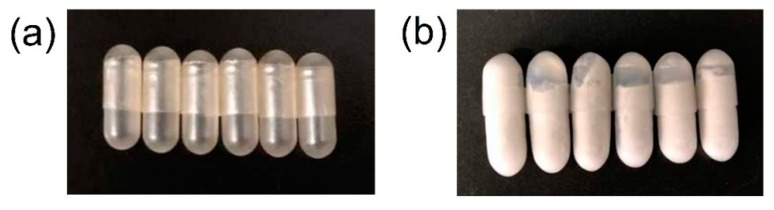
Photographs of (**a**) plant-based enteric hard capsules and (**b**) those after disintegration experiment in simulated gastric juice.

According to the above results, the GG/XG mixing ratio of 7:3 and the plasticizer of PEG-400 were most appropriate for the preparation of plant-based enteric hard capsules, shown in Figure 9a. To study the disintegration properties of plant-based enteric hard capsules, the disintegration experiment of capsules filled with talcum powder was carried out in simulated gastric juice with a pH of 1.5 for 2 h and then in simulated intestinal juice for 30 min. The surface of plant-based enteric hard capsules was smooth and without cracks, and the capsule caps and bodies were not loose and detached after disintegration experiment in simulated gastric juice with a pH of 1.5 for 2 h (Figure 9b). Instead, the capsules completely disintegrated in simulated intestinal juice within 15 min. The transparency of the plant-based enteric hard capsules was good, and the thickness, disintegration time, loss on drying, and heavy metal results met the requirements of China Pharmacopoeia [48].

## 4. Conclusions

The mixture of GG and XG at the ratio of 7:3 was developed as a novel gelling agent to prepare plant-based enteric polymer films. The synergistic interaction between GG and XG was generated by hydrogen bonds of GG and XG, and a plasticizer of PEG-400 was screened out to improve the mechanical properties and water vapor permeability of plant-based enteric polymer films. The results of all the characterization showed that there were the hydrogen interactions between plasticizer PEG-400 and polysaccharides composed of the film. Plasticizer PEG-400 easily inserted between polysaccharide chains constituted the film network, especially GG chains, which produced physical cross-linking between PEG-400 and polysaccharides. The GG/XG mixing ratio of 7:3 and the plasticizer of PEG-400 were the most appropriate for the preparation of plant-based enteric hard capsules met China Pharmacopoeia requirements.

## Figures and Tables

**Table 1:** Rheological properties of solutions at different GG/XG mixing ratios.

GG:XG	Gelling Temperature (°C)	Melting Temperature (°C)	Gelation Rate (×10^−4^ Pa/s)	tanδ_0_
10:0	37.4	55.1	12.7	0.2319
9:1	38.4	60.7	13.4	0.2638
8:2	39.1	62	14.7	0.2627
7.5:2.5	71	60	16.2	0.2458
7:3	82	72	24.1	0.2114
6.5:3.5	67.3	66	24.5	0.2427
6:4	58.9	58	25.0	0.2387
5:5	nd	nd	nd	nd

nd: not detected.

**Table 2:** Activation energy and glass transition temperature of powder mixture, GG film, GG/XG film, and PEG film.

Sample	*E* (KJ/g)	*T*_g_ (°C)
**Powder Mixture**	−90.700	nd
**GG Film**	−116.680	150.1
**GG/XG Film**	−151.610	213.0
**PEG Film**	−123.660	159.5

nd: not detected.
